# Coexpression of CD5 and CD43 predicts worse prognosis in diffuse large B‐cell lymphoma

**DOI:** 10.1002/cam4.1674

**Published:** 2018-07-17

**Authors:** Xiao‐bo Ma, Yan‐ping Zhong, Yan Zheng, Jing Jiang, Yin‐ping Wang

**Affiliations:** ^1^ Department of Pathology First Hospital of Jilin University Changchun China; ^2^ Division of Clinical Epidemiology First Hospital of Jilin University Changchun China

**Keywords:** CD43, CD5, diffuse large B‐cell lymphoma, pathology, prognosis

## Abstract

Both CD5 and CD43 are expressed on the surface of B lymphocytes of definite phase and associated with the adverse outcome in diffuse large B‐cell lymphoma (DLBCL). However, the relationship between CD5 and CD43 expression and the prognostic value of CD5/CD43 coexpression in DLBCL are unknown. We herein determined the correlation between CD5 and CD43 expression, as separate factors or in combination, with the clinicopathological features and survival of 200 patients with DLBCL receiving standard chemotherapy with or without rituximab. Among these DLBCL patients, CD5 expression, CD43 expression, and CD5/CD43 coexpression were detected in 18 (9%), 57 (27%), and 10 (5%) patients, respectively, and all were positively correlated with advanced age and nongerminal cell type. CD5‐positive and CD43‐positive DLBCL patients had poorer event‐free survival (EFS,* P* < 0.001) and overall survival (OS,* P* < 0.001) than CD5‐negative and CD43‐negative patients, respectively. CD5/CD43 coexpression was correlated with a significantly worse prognosis than CD5 or CD43 expression alone. Univariate analysis showed that CD5 expression, CD43 expression, and CD5/CD43 coexpression were all adverse prognostic factors for DLBCL patient survival, and CD5/CD43 coexpression was associated with a greater relative risk for recurrence and death than either CD5 or CD43 expression alone. Multivariate analysis demonstrated that CD5/CD43 coexpression was an independent prognostic factor for EFS (*P* < 0.001) and OS (*P* < 0.001) in DLBCL. In conclusion, our data indicate that DLBCL patients with CD5/CD43 coexpression represent a specific subgroup with a significantly worse prognosis than those expressing either marker alone.

## 1 INTRODUCTION

Diffuse large B‐cell lymphoma (DLBCL) is a group of aggressive non‐Hodgkin's lymphomas with heterogeneous morphology, immunophenotype, molecular abnormality, and clinical behavior.^1^, ^2^ Although the addition of rituximab to the standard chemotherapy regimen (cyclophosphamide, doxorubicin, vincristine, prednisone, and R‐CHOP) makes DLBCL curable in a subgroup of patients, 30% of patients have refractory disease or experience relapse.^3^, ^4^ This suggests high heterogeneity of DLBCL in terms of tumor progression and clinical outcome and highlights the importance of identifying biomarkers to predict the clinical outcome of high‐risk groups or a specific subtype of DLBCL.

The recommended prognostic factors for DLBCL by the International Prognostic Index (IPI) include age >60 years, elevated serum lactate dehydrogenase (LDH), Eastern Cooperative Oncology Group (ECOG) performance status ≥2, stage III or IV, and number of involved extranodal sites >1. However, these five risk factors are not related to the biological features.^5^ According to the results of gene expression profiling studies, DLBCL can be stratified into germinal center B‐cell (GCB)‐like and activated B‐cell (ABC)‐like or non‐GCB‐like subtypes, and DLBCL patients with the ABC subtype have an inferior prognosis,^6^ which is improved by the addition of rituximab to anthracycline‐based regimens. Moreover, increased expression of BCL2 family members plays a role in the resistance of DLBCL to chemotherapy.^7^, ^8^ BCL6 was reported to be associated with a better prognosis, and patients with BCL6‐positive DLBCL experienced relatively favorable outcomes after treatment with the CHOP regimen.^9^ Although these biomarkers may predict the prognosis of DLBCL, few of them have been translated into clinical practice.^10^

CD5 is a cell surface glycoprotein and is typically expressed on T cells and a subset of normal naïve B cells as well as lymphoma cells, mainly chronic lymphocytic leukemia/small lymphocytic lymphoma (CLL/SLL) and mantle cell lymphoma.^11^, ^12^, ^13^ Anatomical localization, gene usage, and function are different between CD5‐positive B cells and CD5‐negative conventional B cells.^14^, ^15^, ^16^ CD5‐positive B cells synthesize immunoglobulin (Ig) M autoantibodies, and increased numbers of CD5‐positive B cells are associated with some types of autoimmune disease.^17^, ^18^, ^19^ CD5 is also expressed in 5%‐10% of de novo DLBCL cases,^20^, ^21^, ^22^ and CD5+ DLBCL has been included as an immunohistochemical subgroup in the fourth edition of the World Health Organization (WHO) classification.^12^ Several studies have demonstrated that DLBCL patients with CD5‐positive expression have a poorer overall survival (OS) rate than those without CD5 expression, regardless of the use of rituximab.^23^, ^24^

CD43 is a multifunctional type I transmembrane protein that regulates multiple cellular functions, such as cell signal transduction, cell adhesion, activation, proliferation, cell survival, and apoptosis.^25^, ^26^, ^27^ CD43 is expressed on the surface of most hematopoietic cells, some lymphomas, and leukemias.^28^ CD43 is also expressed in a variety of solid tumors, but is undetectable in normal tissues and benign lesions.^29^, ^30^ In particular, the expression level of CD43 glycoforms in cancer cells correlates with the progression stage of the disease.^30^ It has been suggested that coexpression of CD43 and CD20 on peripheral B cells is associated with malignancy.^31^ Several studies have showed that CD43 is expressed in approximately 25% of DLBCL cases and is an independent adverse prognostic marker for DLBCL.^32^, ^33^, ^34^

Both CD5 and CD43 have been found to be expressed on the surface of B lymphocytes of definite phase and associated with clinical outcomes of DLBCL patients. It was suggested that combined detection of CD43 and CD5 could differentiate cancer cells from normal T and B cells.^35^ However, to our knowledge, there has been no report about the prognostic value of CD5 and CD43 coexpression in DLBCL. In this study, we analyzed the association between CD5 and CD43 expression alone or in combination with the clinicopathological features and survival of patients with DLBCL.

## 2 MATERIALS AND METHODS

### 2.1 Ethics statement

This study was approved by the Research Ethics Committee of the First Hospital of Jilin University (Changchun, China). All patients provided written informed consent.

### 2.2 Patient cohort

We enrolled 200 Chinese patients with DLBCL diagnosed at the First Hospital of Jilin University from 1 January 2004 to 30 December 2015. The medical records of the patients were retrieved and reviewed. Diagnosis of DLBCL was verified by two professional hematopathologists according to the World Health Organization (WHO) classification system.^2^ Patients who had a history of low‐grade B‐cell lymphoma, human immunodeficiency virus or human T‐cell lymphotropic virus type I infection, primary mediastinal, cutaneous B‐cell lymphoma, or central nervous system were excluded from this study. All patients received either CHOP or R‐CHOP (CHOP plus rituximab) for a median of 5 cycles (3‐8 cycles) and with a median follow‐up time of 62 months (range, 15‐137 months).

### 2.3 Morphological and immunophenotypic analyses

At the time of diagnosis, biopsy samples were collected and processed for hematoxylin and eosin staining. Immunohistochemical analysis was performed using the dextran polymer method (EnVision+; Dako, Glostrup, Denmark) using monoclonal antibodies (mAb) against CD20 (clone BC‐1), CD3 (clone PC3/188A), CD5 (clone CD5/54/F6), CD43 (clone DF‐T1), cyclin D1 (clone 72‐13G), CD10 (clone SP67), Bcl‐6 (clone GI191E/A8), and MUM‐1 (clone MUM1P) from Santa Cruz Biotechnology (Santa Cruz, CA, USA), and Ki‐67 (clone B126.1), FOXP1 (clone JC12), and GCET1 (clone RAM3) from Abcam (Cambridge, UK). Samples were classified as GC (germinal center) or non‐GC (nongerminal center) phenotypes using the Choi algorithm.^36^ A sample was defined as showing immunohistochemical positivity when more than 30% of tumor cells stained positively for CD10 and BCL‐6 or when more than 80% of tumor cells stained positively for MUM1, FOXP1, and GCET1.^36^ To exclude cases of mantle cell lymphoma, CD5+ tissues were further stained for cyclin D1. All the histopathology samples and immunohistochemical analysis were reviewed by two professional hematopathologists.

### 2.4 Statistical analysis

All data were analyzed using SPSS 18.0 software (SPSS Inc., Chicago, IL, USA). Associations between CD5 expression, CD43 expression, CD5/CD43 coexpression, and clinicopathological characteristics were determined using the chi‐square test. Correlation between CD5 and CD43 expression was also evaluated by the chi‐square test. EFS was calculated from the date of diagnosis to the date of documented disease progression, relapse, death, or study termination. OS was calculated from the date of diagnosis until death from any cause or the last follow‐up. EFS and OS were estimated using the Kaplan‐Meier method and compared by the log‐rank test. Univariate analyses, multivariate analyses, and stratified analysis were performed using the Cox proportional hazards regression model. *P* ≤ 0.05 was set as indicative of a significant difference.

## 3 RESULTS

### 3.1 Patient characteristics

The characteristics of the patients including 109 men and 91 women with a median age of 58.0 years (range, 7‐88 years) are summarized in Table 1. Sixty‐seven patients (33.5%) were of the GCB subtype, and 133 patients (66.5%) were of the non‐GCB subtype with 143 (71.5%) with IPI scores of 0‐2 and 57 (28.5%) with IPI scores of 3‐5. The patients in clinical stages I‐II and stages III‐IV were 116 (58%) and 84 (42%), respectively. One hundred and eighteen (59%) were treated with the CHOP regimen, and 82 patients (41%) were treated with R‐CHOP regimen.

**Table 1:** Summary of patient characteristics

Characteristic	Number of patients (%)
Total	200 (100.0)
Age range (years)	7‐88
Median age (years)	58.0
Mean age (years)	54.7
>60	118 (59.0)
≤60	82 (41.0)
Gender	
Male	109 (54.5)
Female	91 (45.5)
Ann Arbor stage	
I‐II	116 (58.0)
III‐IV	84 (42.0)
ECOG PS	
0‐1	158 (79.0)
≥2	42 (21.0)
LDH level	
Normal	75 (37.5)
Elevated	125 (62.5)
Extranodal involvement	
<2	156 (78.0)
≥2	44 (22.0)
B symptoms	
Yes	86 (43.0)
No	114 (57.0)
Bulky tumor^a^	
Yes	39 (19.5)
No	161 (80.5)
IPI	
0‐2	143 (71.5)
3‐5	57 (28.5)
GC phenotype	
GC type	67 (33.5)
Non‐GC type	133 (66.5)
Ki‐67	
<80%	117 (58.5)
≥80%	83 (41.5)
Treatment	
CHOP	118 (59.0)
R‐CHOP	82 (41.0)
Response	
CR	82 (41.0)
PR SD, PD	118 (59.0)

CHOP, cyclophosphamide/doxorubicin/vincristine/prednisone; CR, complete remission; ECOG, Eastern Cooperative Oncology Group; GC, germinal center; IPI, International Prognostic Index; LDH, lactate dehydrogenase; PD, progressive disease; PR, partial remission; PS, performance status; R‐CHOP, rituximab CHOP; SD, stable disease.

a Bulky tumor means the maximum diameter of the tumor is more than 7.5 cm.

### 3.2 Expression of CD5 and CD43 and the correlation between CD5 and CD43 expression in DLBCL

**Figure 1:**
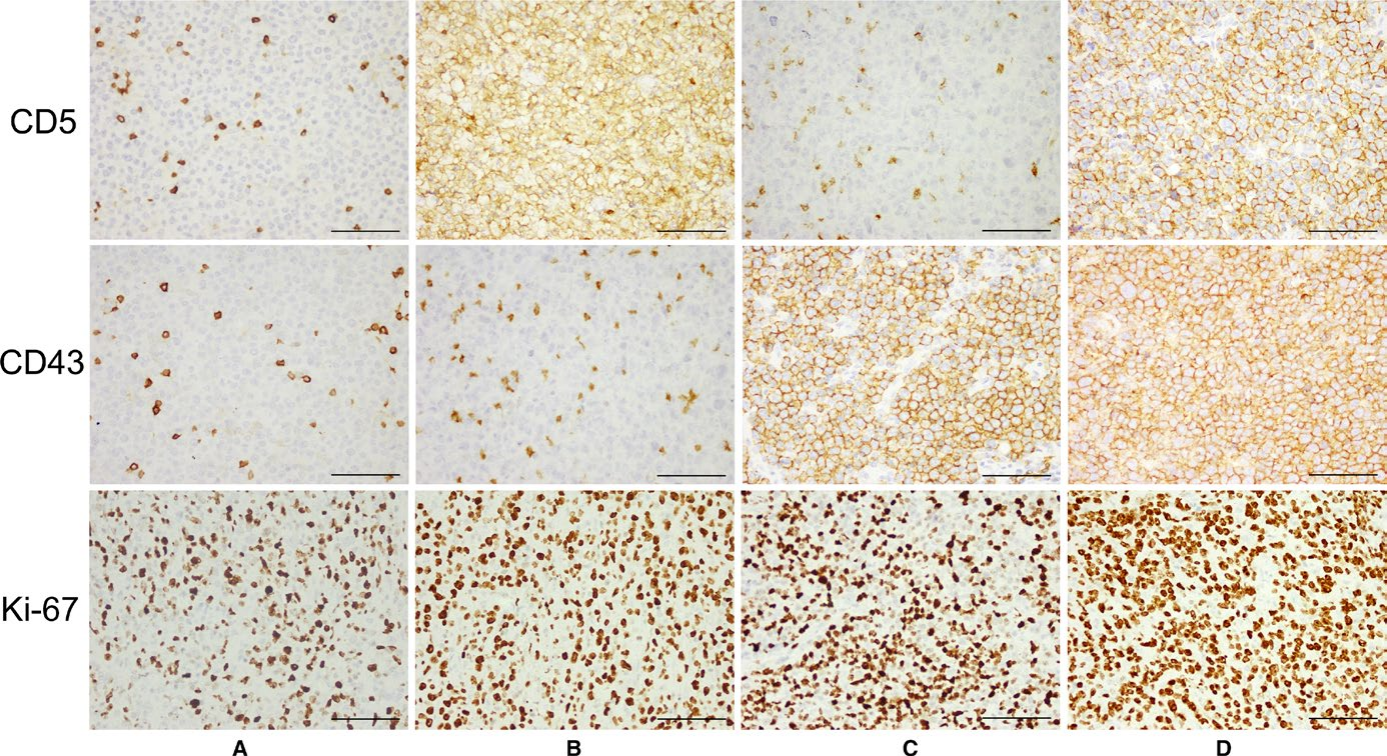
Representative Immunohistochemically Stained Sections of DLBCL Tissues with Different CD5/CD43 Expression Patterns Including CD5−/CD43− (A), CD5+/CD43− (B), CD5−/CD43+ (C), and CD5+/CD43+ (D). The CD5+/CD43+ group showed a relatively higher Ki‐67 index than the other groups. Scale bar = 100 μm (original magnification, ×400)

Expression of CD5 and CD43 in DLBCL tissues was assessed by immunohistochemical staining. Positive staining for CD5 and CD43 expression was detected in 18 (9%) and 54 (27%) cases, respectively; CD5/CD43 coexpression was found in 10 (5%) cases (Table 2). Figure 1 shows the representative immunohistochemical staining profiles for CD5−/CD43− (Figure 1A), CD5+/CD43− (Figure 1B), CD5−/CD43+ (Figure 1C), and CD5+/CD43+ (Figure 1D) in the DLBCL tissues. The expression levels of CD5 and CD43 were significantly correlated due to conegativity (*P* = 0.004, chi‐square test; Table 2). Most expression of CD5 and CD43 was found in overlapping cell populations, and the CD5+/CD43+ group showed a relatively higher Ki‐67 index than the other groups (Figure 1D). Among the 200 DLBCL patients, 8 were positive for CD5 only, 44 were positive for CD43 only, 10 were positive for both CD5 and CD43, and 138 were negative for either CD5 or CD43 expression.

**Table 2:** Correlation between CD5 and CD43 expression

	CD43		Spearman's *R*	χ^2^	*P*
	Positive	Negative			
CD5					
Positive	10	8	0.202	8.183	0.004
Negative	44	138			

### 3.3 Correlation of CD5 and CD43 expression with the clinicopathological characteristics of DLBCL patients

CD5 expression was significantly associated with age >60 years (*P* = 0.045), more extranodal involvement (*P* = 0.006), and non‐GC phenotype (*P* = 0.008). CD43 expression was significantly associated with age >60 years (*P* = 0.004), elevated LDH level (*P* = 0.017), B symptoms (*P* = 0.029), and non‐GC phenotype (*P* = 0.001). CD5/CD43 coexpression was significantly associated with age >60 years (*P* = 0.047), gender (*P* = 0.021), more extranodal involvement (*P* = 0.019), high IPI (*P* = 0.024), non‐GC phenotype (*P* = 0.021), and high Ki‐67 index (*P* = 0.002; Table 3). Therefore, CD5 expression, CD43 expression, and CD5/CD43 coexpression were all positively correlated with advanced age (>60 years) and non‐GC type.

**Table 3:** Correlations between CD5 expression, CD43 expression, and CD5/CD43 coexpression and clinicopathological factors of DLBCL patients

Characteristics	CD5, number (%)			CD43, number (%)			CD5/CD43, number (%)		
	Positive	Negative	*P*	Positive	Negative	*P*	Double Positive	Others	*P*
Overall	18 (9.0)	182 (91.0)		54 (27.0)	146 (73.0)		10 (5.0)	190 (95.0)	
Age (years)									
>60	12 (66.7)	106 (58.2)	0.045	23 (42.6)	95 (65.1)	0.004	7 (70.0)	111 (58.4)	0.047
≤60	6 (33.3)	76 (41.8)		31 (57.4)	51 (34.9)		3 (30.0)	79 (41.6)	
Sex									
Male	11 (61.1)	98 (53.8)	0.555	33 (61.1)	76 (52.1)	0.254	9 (90.0)	100 (52.6)	0.021
Female	7 (38.9)	84 (46.2)		21 (38.9)	70 (47.9)		1 (10.0)	90 (47.4)	
Ann Arbor stage									
I‐II	8 (44.4)	108 (59.3)	0.222	28 (51.9)	88 (60.3)	0.284	4 (40.0)	112 (58.9)	0.237
III‐IV	10 (55.6)	74 (40.7)		26 (48.1)	58 (39.7)		6 (60.0)	78 (41.1)	
ECOG PS									
0‐1	13 (72.2)	145 (79.7)	0.459	38 (70.4)	120 (82.2)	0.068	6 (60.0)	152 (80.0)	0.130
≥2	5 (27.8)	37 (20.3)		16 (29.6)	26 (17.8)		4 (40.0)	38 (20.0)	
LDH level									
Normal	6 (33.3)	69 (37.9)	0.702	13 (24.1)	62 (42.5)	0.017	2 (20.0)	73 (38.4)	0.499
Elevated	12 (66.7)	113 (62.1)		41 (75.9)	84 (57.5)		8 (80.0)	117 (61.6)	
Extranodal involvement									
<2	6 (33.3)	137 (79.1)	0.006	25 (46.3)	118 (80.8)	0.062	3 (30.0)	148 (77.9)	0.019
≥2	12 (66.7)	45 (20.9)		29 (53.7)	28 (19.2)		7 (70.0)	42 (22.1)	
B symptoms									
Yes	8 (44.4)	78 (42.9)	0.897	30 (55.6)	56 (38.4)	0.029	5 (50.0)	109 (57.4)	0.646
No	10 (55.6)	104 (57.1)		24 (44.4)	90 (61.6)		5 (50.0)	81 (42.6)	
Bulky tumor^a^									
Yes	6 (33.3)	33 (18.1)	0.120	15 (27.8)	24 (16.4)	0.072	6 (60.0)	155 (81.6)	0.093
No	12 (66.7)	149 (81.9)		39 (72.2)	122 (83.6)		4 (40.0)	35 (18.4)	
IPI									
0‐2	10 (55.6)	133 (73.1)	0.116	36 (66.7)	107 (73.3)	0.357	4 (40.0)	139 (73.2)	0.024
3‐5	8 (44.4)	49 (26.9)		18 (33.3)	39 (26.7)		6 (60.0)	51 (26.8)	
GC phenotype									
GC type	1 (5.6)	66 (36.3)	0.008	8 (14.8)	59 (40.4)	0.001	0 (0.0)	67 (35.3)	0.021
Non‐GC type	17 (94.4)	116 (63.7)		46 (85.2)	87 (59.6)		10 (100.0)	123 (64.7)	
Ki‐67									
<80%	7 (38.9)	110 (60.4)	0.085	26 (48.1)	91 (62.3)	0.077	1 (10.0)	116 (61.1)	0.002
≥80%	11 (61.1%)	72 (39.6)		28 (51.9)	55 (37.7)		9 (90.0)	74 (38.9)	
Treatment									
CHOP	8 (44.4)	110 (60.4)	0.188	27 (50.0)	91 (62.3)	0.116	4 (40.0)	114 (60.0)	0.210
R‐CHOP	10 (55.6)	72 (39.6)		27 (50.0)	55 (37.7)		6 (60.0)	76 (40.0)	
Response (%)									
CR	4 (22.2)	78 (42.9)	0.090	21 (38.9)	61 (41.8)	0.712	2 (20.0)	80 (42.1)	0.166
PR SD, PD	14 (77.8)	104 (57.1)		33 (61.1)	85 (58.2)		8 (80.0)	110 (57.9)	

CHOP, cyclophosphamide/doxorubicin/vincristine/prednisone; CR, complete remission; ECOG, Eastern Cooperative Oncology Group; GC, germinal center; IPI, International Prognostic Index; LDH, lactate dehydrogenase; PD, progressive disease; PR, partial remission; PS, performance status; R‐CHOP, rituximab CHOP; SD, stable disease.

a Bulky tumor means the maximum diameter of the tumor is more than 7.5 cm.

### 3.4 Prognostic significance of CD5 expression, CD43 expression, and CD5/CD43 coexpression in DLBCL patients

**Figure 2:**
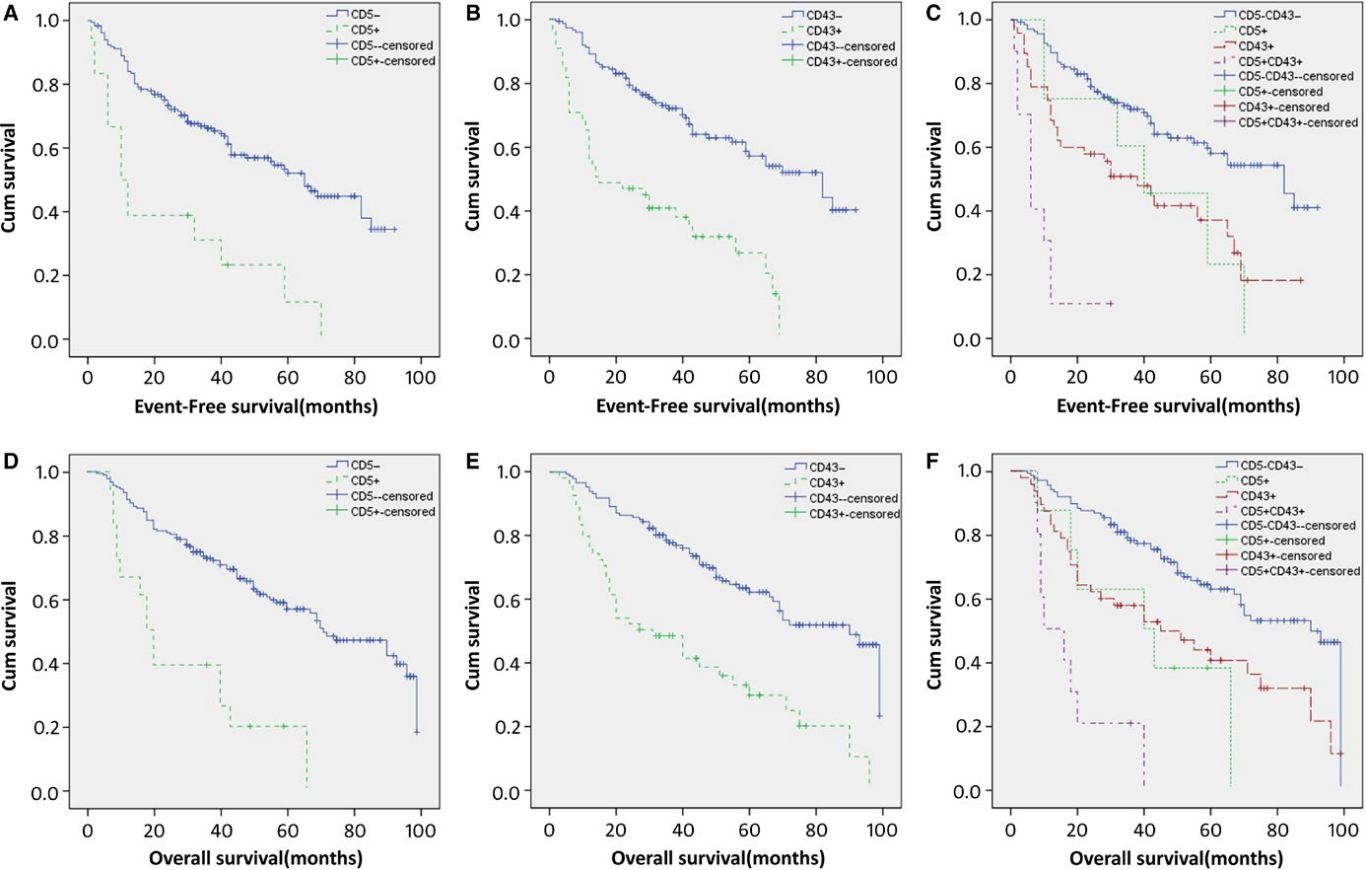
Kaplan‐Meier Survival Curves for CD5 Expression (A, D), CD43 Expression (B, E), and four Groups of CD5 and CD43 Expression (C, F) in DLBCL. Survival was significantly better for patients negative for CD5 (*P* < 0.001 for both EFS and OS) and for CD43 (*P* < 0.001 for both EFS and OS) than that for patients with positive expression level. Patients with the CD5+/CD43+ coexpression profile had the worst outcome for OS among the 4 groups. Pairwise comparisons showed that a statistically significant difference in survival rates existed between the CD5+/CD43+ group and any of the other three groups (*P* < 0.05 for both EFS and OS)

CD5+ DLBCL patients had a significantly poorer EFS (median EFS: 10 months vs 65 months, *P* < 0.001) and OS (median OS: 18 vs 71 months, *P* < 0.001) than CD5− DLBCL patients (Figure 2A,D). The 5‐year EFS rates for patients with CD5+ vs CD5− DLBCL were 11.7% vs 52.1%, and the 5‐year OS rates for patients with CD5+ vs CD5− DLBCL were 19.4% vs 56.2%, respectively. CD43+ DLBCL patients also had significantly poorer EFS (median EFS: 14 months vs 82 months, *P* < 0.001) and OS (median OS: 27 vs 90 months, *P* < 0.001) than CD43− DLBCL patients (Figure 2B,E). The 5‐year EFS rates for patients with CD43+ vs CD43− DLBCL were 25.8% vs 56.6%, respectively, and the 5‐year OS rates for patients with CD43+ vs CD43− DLBCL were 29.2% vs 61.9%, respectively.

To further explore the prognostic significance of CD5/CD43 coexpression, the patients were divided into four groups: CD5/CD43 coexpression (CD5+ and CD43+), CD5 positive only, CD43 positive only, and both negative (CD5− and CD43−). Log‐rank test found that the CD5/CD43 coexpression group had a worse prognosis than either single‐positive group (*P* = 0.004 and 0.024 for the CD5+ group and *P *=* *0.001 and 0.001 for the CD43+ group for EFS and OS, respectively) or the both‐negative group (*P* < 0.001 for both EFS and OS), whereas no difference in survival was found between the CD5+ group and CD43+ group (*P* = 0.536 and 0.921 for EFS and OS, respectively; Figure 2C,F).

### 3.5 CD5 expression, CD43 expression, and CD5/CD43 coexpression are prognostic factors of DLBCL independently of chemotherapy with or without rituximab

**Figure 3:**
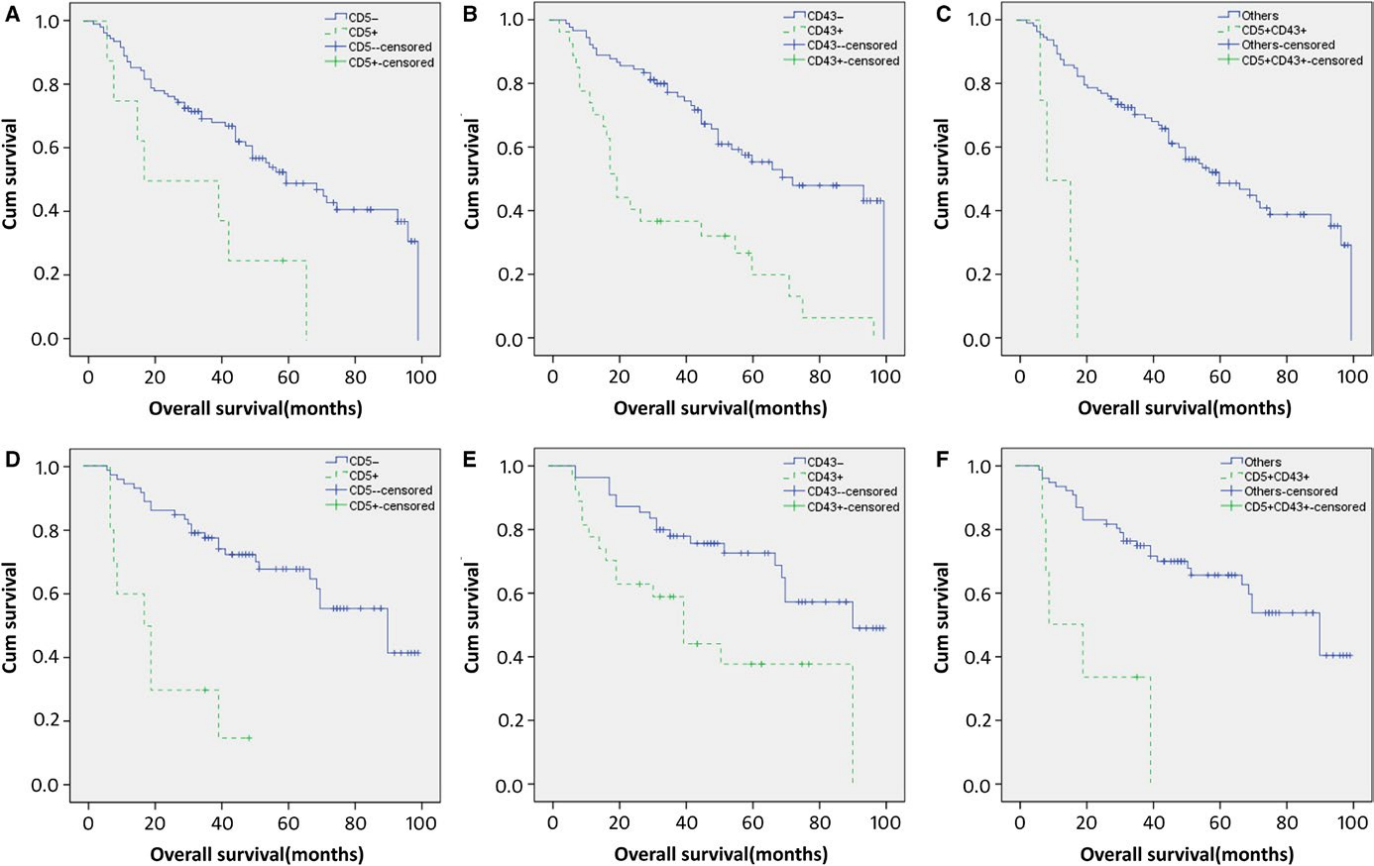
CD5 Expression, CD43 Expression, and CD5/CD43 Coexpression in DLBCL are all Prognostic Factors Independently of Chemotherapy With or Without Rituximab. (A, D) Overall survival according to CD5 expression and chemotherapy with rituximab (D) or without rituximab (A). (B, E) Overall survival according to CD43 expression and chemotherapy with rituximab (E) or without rituximab (B). (C, F) Overall survival according to CD5/CD43 coexpression and chemotherapy with rituximab (F) or without rituximab (C)

To assess whether rituximab treatment affected on the prognostic impact of CD5 expression, CD43 expression, and CD5/CD43 coexpression in DLBCL, the patients were divided into CHOP and R‐CHOP treatment groups. The results showed that CD5 expression, CD43 expression, and CD5/CD43 coexpression were all associated with shorter OS (*P *<* *0.001) in both the CHOP and R‐CHOP groups (Figure 3).

### 3.6 Univariate and multivariate survival analyses

To determine the prognostic value of the clinicopathological factors in combination with CD5 and CD43 expression, univariate Cox regression models were applied. In the univariate survival analysis, advanced age (>60 years), advanced Ann Arbor stage, more extranodal involvement (≥2), elevated LDH, high Eastern Cooperative Oncology Group (ECOG) performance status (PS; ≥2), high IPI, non‐GCB phenotype, high Ki‐67 index (≥80%), CD5 expression, CD43 expression, and CD5/CD43 coexpression were all significant prognostic factors for poor EFS and OS. The relative risk (RR) was estimated by Cox regression. CD5/CD43 coexpression increased the RR for both recurrence and death compared with CD5 or CD43 expression alone (Table 4).

**Table 4:** Prognostic factors that affect EFS and OS (univariate analysis)

Risk factor	Event‐free survival		Overall survival	
	RR (95% CI)	*P*	RR (95% CI)	*P*
Age (>60 vs ≤60 y)	2.888 (1.916‐4.353)	<0.001	2.966 (1.958‐4.491)	<0.001
Sex (female vs male)	0.964 (0.646‐1.437)	0.857	1.017 (0.681‐1.519)	0.935
Ann Arbor stage (III‐IV vs I‐II)	4.106 (2.664‐6.328)	<0.001	4.129 (2.672‐6.380)	<0.001
Extranodal involvement (≥2 vs <2)	3.076 (2.002‐4.725)	<0.001	3.125 (2.034‐4.801)	<0.001
LDH level (evaluated vs normal)	1.962 (1.502‐2.564)	<0.001	2.079 (1.575‐2.744)	<0.001
ECOG PS (≥2 vs <2)	2.673 (1.737‐4.114)	<0.001	2.592 (1.682‐3.995)	<0.001
IPI (3‐5 vs 0‐2)	4.509 (2.891‐7.032)	<0.001	2.390 (1.471‐3.882)	<0.001
GC phenotype (non‐GCB vs GCB)	2.236 (1.388‐3.600)	0.001	3.080 (1.740‐5.452)	<0.001
Treatment (R‐CHOP vs CHOP)	0.759 (0.501‐1.149)	0.192	0.767 (0.506‐1.161)	0.209
Ki‐67 (≥80% vs <80%)	1.691 (1.129‐2.552)	0.011	1.625 (1.085‐2.443)	0.018
CD5 (positive vs negative)	3.300 (1.892‐5.755)	<0.001	3.696 (2.102‐6.499)	<0.001
CD43 (positive vs negative)	3.180 (2.095‐4.829)	<0.001	2.891 (1.916‐4.362)	<0.001
CD5+CD43+ vs others	7.707 (3.742‐15.872)	<0.001	6.250 (3.057‐12.778)	<0.001

CI, confidence interval; RR, relative risk; LDH, lactic dehydrogenase; ECOG PS, Eastern Cooperative Oncology Group performance status; IPI, International Prognostic Index; GC, germinal center; GCB, germinal center B cell.

Clinicopathological factors at the 0.10 level in the univariate analysis, including age, Ann Arbor stage, extranodal involvement, LDH, ECOG PS, non‐GCB phenotype, and CD5/CD43 coexpression, were entered into a multivariate survival analysis model. Treatment was also entered into the model for CHOP and R‐CHOP, which have long been known to be associated with differences in survival. The results demonstrated that advanced age, advanced Ann Arbor stage, evaluated LDH level, treatment without rituximab, and CD5/CD43 coexpression were independent unfavorable prognostic factors for both EFS and OS (Table 5).

**Table 5:** Prognostic factors that affect EFS and OS (multivariate analysis)

Risk factor	Event‐free survival		Overall survival	
	RR (95% CI)	*P*	RR (95% CI)	*P*
Age (>60 vs ≤60 y)	2.276 (1.429‐3.625)	0.001	2.417 (1.523‐3.836)	<0.001
Ann Arbor stage (III‐IV vs I‐II)	2.768 (1.672‐4.584)	<0.001	3.011 (1.810‐5.008)	<0.001
Extranodal involvement (≥2 vs <2)	1.435 (0.877‐2.350)	0.151	1.325 (0.803‐2.186)	0.271
LDH level (evaluated vs normal)	1.808 (1.027‐3.185)	0.040	1.911 (1.081‐3.378)	0.026
ECOG PS (≥2 vs <2)	1.439 (0.878‐2.356)	0.148	1.396 (0.861‐2.263)	0.176
GC type (non‐GCB vs GCB)	1.277 (0.762‐2.139)	0.354	1.414 (0.842‐2.373)	0.190
Treatment (R‐CHOP vs CHOP)	0.575 (0.372‐0.889)	0.013	0.549 (0.356‐0.848)	0.007
Ki‐67 (≥80% vs <80%)	1.267 (0.824‐1.948)	0.280	1.220 (0.789‐1.888)	0.371
CD5+CD43+ vs others	7.026 (3.167‐15.586)	<0.001	7.300 (3.250‐16.396)	<0.001

CI, confidence interval; RR, relative risk; LDH, lactic dehydrogenase; ECOG PS, Eastern Cooperative Oncology Group performance status; IPI, International Prognostic Index; GC, germinal center; GCB, germinal center B cell.

The associations between CD5/CD43 coexpression and DLBCL survival were further evaluated by a stratified analysis of age, Ann Arbor stage, LDH level, and treatment. As shown in Table 6, the prognostic value of CD5/CD43 coexpression was independent of any of these factors. Moreover, the adverse effect of CD5/CD43 coexpression was more prominent in patients with a normal LDH level (adjusted RR = 42.557 for EFS, adjusted RR = 10.736 for OS).

**Table 6:** Stratified analysis for CD5/CD43 coexpression and DLBCL patients’ survival

Risk factor	Event‐free survival		Overall survival	
	Adjusted RR (95% CI)	*P*	Adjusted RR (95% CI)	*P*
Age				
≤60 y	10.964 (3.458‐34.760)	<0.001	12.626 (3.726‐42.783)	<0.001
>60	12.125 (4.347‐33.821)	<0.001	9.099 (3.307‐25.036)	<0.001
Ann Arbor stage				
I‐II	10.566 (3.016‐37.013)	<0.001	7.083 (2.048‐24.501)	<0.001
III‐IV	7.210 (2.897‐17.944)	<0.001	8.023 (3.119‐20.635)	<0.001
LDH level				
Normal	42.557 (5.871‐308.476)	<0.001	10.736 (2.260‐50.992)	<0.001
Evaluated	4.838 (2.166‐10.806)	<0.001	5.644 (2.489‐12.800)	<0.001
Treatment				
CHOP	7.656 (2.619‐22.385)	<0.001	9.081 (3.065‐26.904)	<0.001
R‐CHOP	8.280 (3.027‐22.648)	<0.001	5.276 (1.969‐14.138)	0.001

CI, confidence interval; RR, relative risk; LDH, lactic dehydrogenase.

## 4 DISCUSSION

The present study demonstrated that the expression rates of CD5 and CD43 in DLBCL were 9% and 27%, respectively. Either CD5 or CD43 expression was correlated with advanced age (>60 years), evaluated LDH, B symptoms, non‐GC phenotype, and DLBCL mortality. Moreover, in comparison with those for CD5− and CD43− patients, the OS and EFS rates for CD5+ and CD43+ patients were significantly lower. Our data further support the previous observation that either CD5 or CD43 expression can predict poor prognosis in DLBCL.^20^, ^32^, ^34^, ^37^, ^38^

The simultaneous expression of CD5 and CD43 on the surface of B lymphocytes of definite phase has led to the suggestion that combined detection of CD43 and CD5 might differentiate cancer cells from normal T and B cells.^35^ To test whether there is any correlation between CD5 and CD43 expression in DLBCL, we performed chi‐square test and found that the expression of CD5 and CD43 was strongly associated with most of them expressed in overlapping cell populations. Further analysis of the correlation of CD5/CD43 coexpression with the clinicopathological characteristics of DLBCL showed that CD5/CD43 coexpression was significantly associated with advanced age, gender (male), more extranodal involvement, high IPI, high Ki‐67 index, non‐GC phenotype, and DLBCL mortality. Survival analysis showed that the patients with CD5/CD43 coexpression had a significantly worse prognosis than either single‐positive group or both‐negative group. CD5/CD43 coexpression increased the RR for both recurrence and death compared with CD5 or CD43 expression alone. Moreover, we found that CD5 expression, CD43 expression, and CD5/CD43 coexpression were all significantly associated with the non‐GC phenotype, and the patients with CD5/CD43 coexpression were all non‐GC phenotype. Our results suggest that CD5/CD43 coexpression cases may represent a specific subset of DLBCL with even more inferior prognosis.

CD5+CD43+ DLBCL may be derived from CD5+CD43+ B cells during B‐cell development or the malignant transformation of normal B cells. B cells are divided into conventional B (B‐2) and B‐1 cells, with B‐1 cells predominantly produced during fetal and neonatal development.^39^, ^40^, ^41^ In mice, B‐1 cells are further classified into B‐1a and B‐1b cells with B‐1a cells expressing CD5. CD5 is also expressed on regulatory B cells that involve in immune evasion.^42^, ^43^ CD43 has been shown to be expressed on early hematopoietic cells derived from human embryonic stem cells (hESCs), pro‐B, and early‐stage pre‐B cells including B‐1 and B‐2 cells, which have lost CD43 expression in late stage.^44^, ^45^ Some earlier studies in mice indicated that B‐1a cells may be the origin of some B‐cell lymphomas.^46^, ^47^ Coexpression of CD5/CD43 can only be found in B‐1a cells in mice, which appear early in B‐cell development and have many functions of primitive cells. Although human CD5+CD43+ B cells have not been clearly characterized and whether these cells are equivalent to B‐1a cells in mice is unclear, some studies support the hypothesis that a subset of B cells possessing the characteristics of B‐1a cells of mice exists in humans.^48^, ^49^, ^50^, ^51^ These B‐1a‐like cells may be the origin of the CD5+CD43+ cells in DLBCL, which warrants further investigation.

CD5 and CD43 may be involved in the pathogenesis of DLBCL through a variety of mechanisms. CD5 can promote B‐cell survival through autocrine production of interleukin (IL)‐10.^52^ In addition, CD5 maintains B‐cell survival by modifying intracellular calcium mobilization and modulating various signaling pathways including ERK1/2, PI3K, and calcineurin.^53^ It was also reported that CD5+ DLBCL cases often have complex chromosomal aberrations,^54^ suggesting a potential role for CD5 in the regulation of chromosome stability in B cells. CD43 has been shown to play an important role in cell cycle progression, apoptosis, and immune response of B lymphocytes.^55^, ^56^ CD43 also was reported to modulate cell‐to‐cell adhesion between hematopoietic cells.^27^ Moreover, CD43 can abrogate contact inhibition of cell growth through a molecular mechanism that involves AKT‐dependent Merlin phosphorylation and degradation.^57^ A mouse study suggested that anti‐UN1/CD43 antibody has antitumor activity in UN1‐positive HPB‐ALL lymphoblastoid T cells.^58^ Consistently, a recent study showed that patient‐derived antibody recognizes a unique CD43 epitope expressed on all AML and has antileukemia activity in mice.^59^ However, the mechanism of the coexpression CD5 and CD43 in the initiation, progression, and maintenance of DLBCL is currently unknown and needs further investigation.

Although our results demonstrate the significance of CD5/CD43 coexpression on multivariate analysis, there are some weaknesses in our study. In particular, there were only 10 cases with CD5/CD43 coexpression in our study, and thus, even smaller case numbers were available when patients were separated based on the CHOP or R‐CHOP regimen. This low case number may increase the bias in our study. Therefore, the prognostic significance observed in the present study needs to be validated by future independent studies.

In summary, DLBCL with CD5/CD43 coexpression may be a clinicopathological variant of DLBCL. However, the origin of CD5/CD43‐coexpressing B cells and the precise mechanisms by which coexpression of CD5/CD43 alters the behavior of DLBCL are unclear. Given that the majority of DLBCL patients with CD5/CD43 coexpression are older with a poor performance status and that the addition of rituximab to routine chemotherapy does not improve the outcomes of these patients, it will be important to better characterize this subset of DLBCL.

## CONFLICT OF INTEREST

The authors have no conflict of interest to declare.
